# Myocontrol is closed-loop control: incidental feedback is sufficient for scaling the prosthesis force in routine grasping

**DOI:** 10.1186/s12984-018-0422-7

**Published:** 2018-09-03

**Authors:** Marko Markovic, Meike A. Schweisfurth, Leonard F. Engels, Dario Farina, Strahinja Dosen

**Affiliations:** 1 Applied Rehabilitation Technology Lab (ART-Lab), Department of Trauma Surgery, Orthopedics and Plastic Surgery, University Medical Center Göttingen, Georg-August-University, 37075 Göttingen, Germany; 2 Faculty of Life Sciences, Hochschule für Angewandte Wissenschaften Hamburg, Ulmenliet 20, 21033 Hamburg, Germany; 3 Biorobotics Institute, Scuola Superiore Sant’Anna, Viale R. Piaggio, 34, 56025 Pontedera, PI Italy; 4 Neurorehabilitation Engineering Department of Bioengineering Imperial College London, London, SW7 2AZ UK; 5 Faculty of Medicine, Department of Health Science and Technology, Center for Sensory-Motor Interaction, Aalborg University, DE-9220 Aalborg, Denmark

**Keywords:** Myoelectric prosthesis, Baseline, Routine grasping, Grasping force, Sensory feedback, Closed-loop control

## Abstract

**Background:**

Sensory feedback is critical for grasping in able-bodied subjects. Consequently, closing the loop in upper-limb prosthetics by providing artificial sensory feedback to the amputee is expected to improve the prosthesis utility. Nevertheless, even though amputees rate the prospect of sensory feedback high, its benefits in daily life are still very much debated. We argue that in order to measure the potential functional benefit of artificial sensory feedback, the baseline open-loop performance needs to be established.

**Methods:**

The myoelectric control of naïve able-bodied subjects was evaluated during modulation of electromyographic signals (*EMG task*), and grasping with a prosthesis (*Prosthesis task*). The subjects needed to activate the wrist flexor muscles and close the prosthesis to reach a randomly selected target level (routine grasping). To assess the baseline performance, the tasks were performed with a different extent of implicit feedback (proprioception, prosthesis motion and sound). Finally, the prosthesis task was repeated with explicit visual force feedback. The subjects’ ability to scale the prosthesis command/force was assessed by testing for a statistically significant increase in the median of the generated commands/forces between neighboring levels. The quality of control was evaluated by computing the median absolute error (MAE) with respect to the target.

**Results:**

The subjects could successfully scale their motor commands and generated prosthesis forces across target levels in all tasks, even with the least amount of implicit feedback (only muscle proprioception, EMG task). In addition, the deviation of the generated commands/forces from the target levels decreased with additional feedback. However, the increase in implicit feedback, from proprioception to prosthesis motion and sound, seemed to have a more substantial effect than the final introduction of explicit feedback. Explicit feedback improved the performance mainly at the higher target-force levels.

**Conclusions:**

The study establishes the baseline performance of myoelectric control and prosthesis grasping force. The results demonstrate that even without additional feedback, naïve subjects can effectively modulate force with good accuracy with respect to that achieved when increasing the amount of feedback information.

## Background

The hands are an essential part of our body and our most important tool to interact with the world. Not only do we grasp, move, and explore objects using the hands, but they also contribute to our interaction and communication with other living beings through the language of gestures. This refined control of the hands is due to a seamless integration of feedforward motor commands and an elaborate network of sensory feedback (i.e., touch, temperature, nociception, proprioception, kinesthetic feedback) we receive [1].

The loss of a hand in people with transradial amputation can have a pronounced effect on the performance of daily-life activities and the general quality of life. The state-of-the-art in recovering hand function in persons with transradial amputation is to equip them with myoelectric prostheses. These systems are controlled through the activity of the hand and wrist flexor and extensor muscles in the residual limb, recorded by surface electromyography (sEMG). This is a robust and intuitive control scheme for simple, single degree of freedom grippers, since the muscles that were originally used to control the hand and wrist are now employed to open (extensors) and close (flexors) the prosthesis. However, the restoration of lost functions is only partial, as commercially available myoelectric prostheses do not provide explicit sensory feedback about the prosthesis state. The only exception is a recently presented system [2] providing a simple feedback of touch onset using a single vibration motor embedded in the distal part of the prosthesis.

Sensory integration is crucial for motor execution, adaptation and learning [3]. Therefore, as long as no sensory feedback is provided, prosthetic hands are believed to remain suboptimal assistive devices, rather than full bionic hand replacements [4]. Researchers have implemented different methods to provide reliable, intuitive feedback to the user [5]. Most solutions follow the general approach known as sensory substitution [6], in which an alternative sensory modality is used to compensate for the lost sense. In this, the prosthesis is equipped with sensors measuring the system state (joint angles) and interaction with the environment (grasping force), and the sensor data are transmitted to the user through electrical or mechanical stimulation eliciting tactile sensations [7]. The feedback information is coded by changing stimulation parameters, intensity and/or frequency (parameter modulation) and/or location (spatial modulation). In electrical stimulation, low-intensity electrical current pulses are delivered to the skin through surface electrodes, activating superficial skin afferents [8], or directly to the peripheral nerves [9] and/or brain [10] using implantable interfaces. The most common method to deliver direct mechanical stimulation, on the other hand, is to use vibration motors [7]. These can be simple pager vibrators [11–13] with a single input controlling both intensity and frequency, or more advanced voice-coil devices [14], which can modulate the stimulation parameters independently (two control inputs). The prosthesis grasping force was considered most often as the variable to feed back [5], as it cannot be easily assessed visually (contrary to joint angles, for example).

However, even in the absence of explicit force feedback from the prosthesis, the control is not completely “open-loop” since the users can still rely on incidental information sources. First, the prosthesis responds proportionally, that is, the stronger the contraction of the user muscles, the faster the velocity of closing and hence the higher the resulting contact force when grasping. Therefore, the user can rely on the proprioceptive feedback from the remaining muscles (sense of contraction) to control the force. In addition, the user can exploit visual feedback to estimate and adjust the velocity of closing, and thereby indirectly and predictively the resulting grasping force [15]. This strategy can be facilitated by additional cues, such as the sound from the motor and the perception of vibrations transmitted through the socket. Finally, humans are capable of internalizing the dynamics of the system they are controlling [16, 17]. They can use these internal models to operate the system predictively through precomputed feedforward commands. This process translates, at least partly, to the control of prosthetics [18]. For example, it was shown that prosthesis users are able to scale the applied grasping forces in an anticipatory manner depending on the perceived state of the target object (fragile vs. rigid) [19].

Prosthesis users can exploit implicit feedback sources in their daily use of the prosthetic device [20, 21]. However, the quality of control achievable by using incidental feedback has never been systematically investigated. This assessment is important because it represents the baseline performance of “open-loop” control. Such baseline can then be used as a reference to evaluate explicit feedback strategies. This could contribute to clarifying the role of feedback and to quantifying the benefits of closing the loop in upper-limb prosthetics, especially because there is no unanimous agreement whether and to what extent explicit feedback is functionally useful for prosthesis control. Studies on the topic are indeed often contradictive [14, 22–24] and/or inconclusive [25].

In most studies, a specific solution was presented, and the prosthesis performance with feedback was compared to that without the feedback. These are important developments that can demonstrate the effectiveness of a particular feedback interface. However, such studies do not explicitly reveal the reasons why the presented feedback improved or failed to improve the performance. To address the latter, basic studies in controlled conditions should be performed to investigate the general nature of the mechanisms governing closed-loop control in prosthetics. The present study aims at closing that gap in the literature by investigating the role of incidental and explicit feedback sources in prosthesis control. We first determined the “open-loop” baseline and then compared it to the performance achieved using ideal explicit feedback. The subjects’ task was to control the magnitude of muscle activation and prosthesis grasping force. In the baseline condition, the subjects relied on natural proprioception from their muscles plus visual and audio cues related to prosthesis movement, whereas in the explicit feedback condition, the information on the generated grasping force was shown on a computer screen. The aim was to assess the impact of different amounts of implicit feedback on prosthesis force control and to investigate if and how much the performance can be improved by supplementing the implicit sources with explicit force information.

## Materials and methods

### Ethics and consent

Ten able-bodied subjects (22 ± 3 yrs., 6 men, 4 women) participated in the experiment. Subjects were informed about the experiment both in writing and orally. The study was approved by the Ethics committee of the University of Göttingen (04/2016). All experiments were conducted in accordance with the declaration of Helsinki, and all participants provided written informed consent prior to participation in the experiments.

### Experimental setup

The experimental setup consisted of: 1) a Michelangelo hand controlled proportionally using a single dry EMG electrode with embedded amplification, filtering and rectification circuit (13E200, Otto Bock Healthcare GmbH, Vienna, AT); 2) a wooden block to be grasped by the prosthesis; and 3) a standard desktop computer with a 22″ screen. The control-loop was implemented in Matlab Simulink 2015b (MathWorks, US) using a flexible test bench for the assessment of the closed-loop human manual control [26] and executed on the host PC in real time at 100 Hz. Since it has an integrated rectification and filtering circuit, the EMG electrode outputs the envelope of the acquired EMG (smoothed signal). The provided EMG envelope was sampled at 100 Hz by the embedded prosthesis controller and sent to the host PC via a Bluetooth link, where it was additionally filtered using a 2nd order Butterworth low-pass filter with a cutoff frequency of 2 Hz. Visual feedback for the subject was displayed on the computer screen. The average Bluetooth communication latency was around 80 ms.

**Fig. 1:**
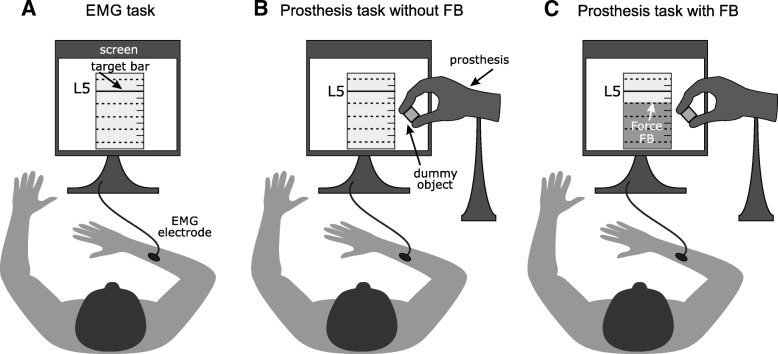
Setup in EMG task (**a**), Prosthesis task without feedback (**b**), and Prosthesis task with feedback (**c**). The desired level of normalized muscle activation (EMG task) or prosthesis force (Prosthesis tasks) was shown on the computer screen (full line, L5). All other levels were indicated with dashed lines. The lowest level was not used as target level (see text for further explanation). In the Prosthesis task without feedback, the subjects could see and hear the prosthesis but did not see the level of generated force. In the Prosthesis task with feedback, they additionally received visual feedback on the generated force (bar in **c**)

The subjects sat comfortably in front of a desk with the computer screen positioned approximately 75 cm away from them (Fig. 1). The prosthesis was securely fixed to the surface of the table, between the subject and the computer monitor. Therefore, the subjects had a clear view of the prosthesis and could also hear the motor sound, with the peak frequencies in the range from 170 Hz to 500 Hz corresponding to different prosthesis closing speeds (20–100% of maximum speed). A rigid object was fixed to the prosthesis’ thumb, so that when the prosthesis closed, it grasped the object. Prior to the experiment, the optimal placement for the EMG electrodes was determined by palpating the ventral aspect of the forearm during wrist/hand flexion movement. The skin was prepared with a small amount of abrasive gel (everi, SpesMedica, IT), and an elastic band was used to strap the electrode firmly to the forearm. The subjects kept their arm in a comfortable semi-upright position, resting their elbow on a cushion placed on the desk and maintained the same position throughout the experiment. Importantly, the subjects’ wrist was left free and unimpaired during the experiment. The analog electrode gain was adjusted so that the signal fluctuated around 85% of the amplifier saturation level when the subjects performed maximum muscle activation, exploiting thereby the full range of the analog amplifier.

### Experimental tasks

The experiment comprised three parts in which the subjects controlled two output variables, namely, the level of muscle contraction (*EMG task*) or the level of grasping force when controlling the prosthesis (*Prosthesis task*). The grasping force was controlled in two tasks, first without (*Prosthesis task without feedback*) and then with an explicit visual force feedback shown on the computer screen (*Prosthesis task with feedback*). The generated and reference muscle activations and prosthesis forces were all normalized to the interval [0%, 100%], as explained below.

**Fig. 2:**
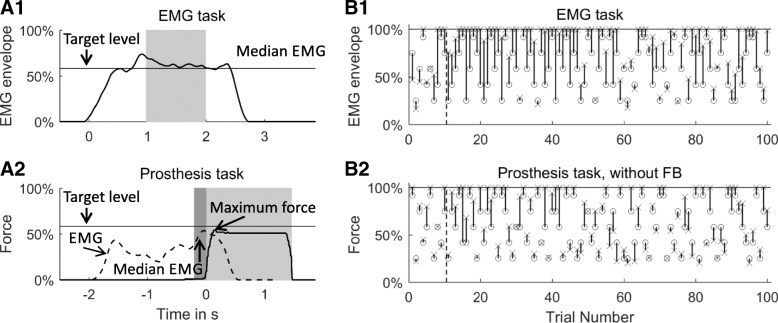
**a** Example time course of the generated signals during a trial in the EMG task (a1) and in the Prosthesis Task (a2). The time windows used for computing the trial outcomes are marked, in the EMG task being the median EMG (a1, light grey) and in the Prosthesis tasks being the maximal force extraction and the median EMG before touch onset (a2, light and dark grey, respectively). **b** Exemplary sequence of target levels (black circles) and generated myoelectric commands and prosthesis forces (crosses) in the EMG task (b1) and the Prosthesis task without feedback (b2). The median absolute error in each trial (length of the line) was used for performance analysis. The first ten trials (indicated by the vertical dashed black line) were regarded as the familiarization phase of the task and thus excluded from the analysis

In the *EMG task* (Fig. 1a), the prosthesis was turned off, and no feedback was provided during the task. The subjects were asked to contract their hand/wrist flexor muscles (the hand was not) to reach a given target level and to maintain the contraction until the trial counter expired (2 s, Fig. 2a1). After each contraction, the subjects had to relax the muscles to initiate a new trial. The filtered myoelectric command was used as the momentary estimate of the muscle activation level. To normalize the signal, the maximum activation was measured as the strongest contraction that the subjects could maintain for 2 s without fatiguing. The muscle signal maximum was defined as the average of five measurements. Finally, the muscle activation signal was normalized by linearly mapping the range between 10 and 100% of the obtained maximum to the interval [0%, 100%]. A myoelectric prosthesis generally operates proportionally, and the level of muscle activation is therefore translated into the magnitude of grasping force. Therefore, the role of the EMG task was to assess how well the subjects could generate the commands to the prosthesis (i.e., muscle-activation levels) by relying only on the natural proprioception from their own muscles (sense of contraction).

In both prosthesis tasks (Fig. 1b-c), the subjects were asked to close the prosthesis using the power grip (all fingers) so that a given target level of force was generated upon contacting the object. The task was performed by contracting the hand/wrist flexor muscles to close the prosthesis from the fully opened position. While the EMG calibration remained the same as in the *EMG task*, the force was normalized with respect to the maximum prosthesis force (~ 100 N). The myoelectric signal proportionally controlled the velocity of closing and the resulting grasping force. The prosthesis input-output function was rather linear, so that the normalized myoelectric input produced approximately the same magnitude of the normalized grasping force (i.e., X % of EMG led to X % of force). The subjects were instructed to activate the flexor muscles to the level that they estimate would lead to the desired grasping force and then maintain the contraction until the prosthesis closed (Fig. 2a2). Therefore, the subjects performed virtually the same protocol as in the EMG task, but this time they could observe the prosthesis response, that is, closing motion as well as motor sound. Since the prosthesis was non-backdrivable and the subjects controlled only the prosthesis closing (using a single EMG channel), the force could not be decreased after contact. When the prosthesis closed around the object, the subject could relax his/her muscles and the prosthesis opened automatically 1.75 s after touch onset.

In the *Prosthesis task without feedback* no explicit force feedback was given (Fig. 1b). The aim was to assess if the subjects would generate better myoelectric commands than in the *EMG task*, by activating the muscles more consistently and accurately, if they received additional incidental feedback (prosthesis motion and sound plus muscle proprioception). Here, the subjects were instructed to focus and rely on all the feedback cues available. In addition, the forces generated in this test established the baseline of force control with no explicit force feedback.

In the *Prosthesis task with feedback* the measured maximum force was shown on the computer screen, revealing to the subject the force that they were generating in that trial (Fig. 1c). The aim was to assess if the subjects would further improve the generation of myoelectric commands and/or the quality of force control, if they were provided with explicit feedback on the generated forces. The subjects were instructed to follow the same strategy as in the two previous tasks, that is, to close the hand using one continuous contraction so that the generated force was produced directly upon contacting the object, without the need to steer the force once the hand was closed. Therefore, the visual feedback was not used to modulate the muscle activation (prosthesis force) during an ongoing trial. Instead, the feedback provided the outcome of the present trial (generated grasping force) and the subject used this information to adjust the muscle activation in the next grasp. Again, the subjects were instructed to focus and rely on all available feedback cues.

### Experimental protocol

The tasks were ordered according to the increasing amount of feedback information, that is, the subjects first performed the *EMG task* (proprioception), followed by the *Prosthesis task without feedback* (proprioception, prosthesis), and finally the *Prosthesis task with feedback* (proprioception, prosthesis, force). This sequence was chosen so that, in each new task, the subjects were provided with additional feedback sources, thereby preventing unwanted across-task learning effects [18], which could potentially mask the impact of less feedback on performance. In each trial, the aim was to produce the indicated level of muscle activation (*EMG task*) or force (*Prosthesis tasks*), as explained in the previous section. The full signal range [0%, 100%] was divided into six equal intervals, and the middle points of the intervals, from the second to the sixth, were adopted as the five target levels (upper five dashed lines, Fig. 1). It should be noted that, due to the inherent coupling mechanism in the prosthesis, generating a command to close the prosthesis at the minimum velocity consistently produced forces within the 2^nd^ interval (see [27] for a comparable discussion). This is the reason why the first interval was not considered as target. The subjects were informed about that coupling, and as will be discussed in the following paragraph, they knew how to exploit it. The trials were organized into 20 sequences of 5 trials, and within each sequence, all target levels appeared once in a randomized order (Fig. 2b1 and b2). Therefore, there were 100 trials per task, with 20 trials per target level (out of which the last 18 were used for analysis, see section Data Analysis). To prevent fatigue, the subjects took a 2-min break after 50 trials. In each trial, the target level was indicated on the computer screen, textually (L2 to L6) and as a horizontal line on the vertical bar (Fig. 1a).

Before starting the *EMG task*, the subjects were trained on how to produce the maximum (100%) and minimum value (just above zero) of the myoelectric command. To this aim, they were provided with visual feedback of the myoelectric command and asked to generate the respective values for several consecutive trials. Once they were sufficiently accurate in reaching them, they repeated the same task with eyes closed. The training was finished when they could generate the maximum/minimum myoelectric command five times in a row without relying on the visual feedback. At this point, it was assumed that the subjects had calibrated their control of muscle activation (*EMG task*). The subjects then performed the sequence of grasping trials. They were instructed to use the learned minimum/maximum values as a reference to scale their motor commands to the target level indicated in the trials. Importantly, before starting the *Prosthesis task* the subjects were introduced in detail to the basics of the prosthesis operation, including proportional control, the linear relation between closing velocity and generated force, and the non-backdrivability. The prosthesis closing and force control was demonstrated by closing it, via artificially-generated signals, at the maximum and minimum velocity for several times. The subjects were also allowed to close the prosthesis five times without receiving explicit force feedback, in order to get familiar with its control.

### Data analysis

The trial outcome in the *EMG task* (generated myoelectric command) was the median of the generated muscle activation, computed over the last 1-s window of the 2-s trial duration (Fig. 2a1). In the *Prosthesis tasks*, the maximum of the generated grasping force (generated force) and the median EMG in the last 200 ms before touch onset (generated myoelectric command) were used as trial outcomes (Fig. 2a2). The time offset of 200 ms was introduced because of the prosthesis’ mechanical inertia; that is, the time delay for building up the grasping force in response to a given EMG command. To account for the subjects’ familiarization with the experimental condition, the first ten trials of each task were excluded from the analysis. The Kolmogorov-Smirnov tests showed that the data were not normally distributed; consequently, we used non-parametric tests for statistical analysis and median and interquartile range to report the results. The significance level was set to a Type-I error level of 0.05 (*p* < 0.05) in all tests described below.

#### Scaling across target levels

The goal of this analysis was to evaluate, separately for each of the three tasks, if the subjects could successfully scale their myoelectric commands and generated prosthesis forces according to the indicated target levels. To this aim, the medians of the generated muscle activations and prosthesis forces were determined for each target level and subject and statistically compared across target levels within the same task. A Friedman test was applied to evaluate statistically significant differences between levels 2 to 6. If present, Bonferroni-Holmes-corrected Wilcoxon signed-ranks tests were used to assess statistically significant differences between the neighboring levels (four tests per Friedman test). The analysis revealed how many statistically different levels of myoelectric commands and prosthesis forces the subjects could generate. In the ideal case, this would be equal to the number of target levels (five).

#### Performance between tasks

**Fig. 3:**
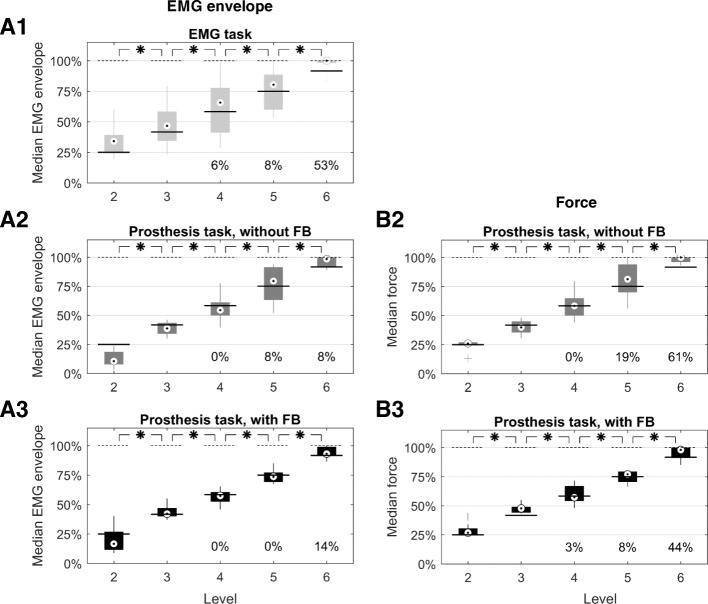
Distribution of generated muscle activations (a1 to a3) and prosthesis forces (b2 to b3) for each target level. For the *EMG task* (a1, light grey) and the *Prosthesis task without *(a2, b2, dark grey) and *with *(a3, b3, black) *feedback*, the distribution of the (per-subject) median of muscle activations (**a**) / forces (**b**) is visualized using boxplots, depicting the overall median (circle), interquartile range (box), maximal/minimal values (lines) and outliers (pluses). Black continuous lines denote the target levels, black dashed lines the level of saturation. For each task, significant differences between the muscle activations / forces generated while aiming at the neighboring target levels are marked with an asterisk (*p* < 0.05, Bonferroni-Holmes corrected). For levels 4 to 6, the median percentage of saturations per subject is given

Myoelectric command generation was analyzed separately from force generation. The goal of this analysis was to assess if and how the amount of feedback altered the reproducibility of myoelectric commands and consequently the quality of force generation. To gain a more detailed insight into the impact of feedback in prosthesis force control, a level-wise force analysis was performed between the two *Prosthesis tasks (with* vs. *without explicit feedback)*. The exact procedures for these two analyses are described in the remainder of this paragraph. Levels 2 and 6 were excluded as the prosthesis control was very different from the other levels. Namely, when aiming at level 6, the subjects often saturated both EMG command as well as the prosthesis force (normalized myoelectric commands and forces were close to 100%). Therefore, the true distribution of the generated myoelectric commands and prosthesis forces at level 6 could not be captured. For the target level 2, the subjects exploited the coupling inherent in the prosthesis, as explained in the section Experimental Protocol. They realized that to reach the 2nd level, they simply needed to produce the minimal myoelectric command to close the prosthesis at the minimum velocity, as the prosthesis could not generate lower forces. Therefore, they consistently undershot in the produced EMG (see Fig. 3a2 and a3). For the remaining levels 3 to 5, the median absolute error (MAE), defined as the absolute difference between the generated and desired muscle activation/prosthesis force, was calculated to assess the performance. The MAE was determined separately for each task (also for myoelectric command and force in the *Prosthesis task*), target level, and subject. To perform an overall analysis, the mean MAE across target levels was calculated for each subject and task. A Friedman test was applied to compare the overall performance in myoelectric command generation between the three tasks. If the test indicated significant differences, the tasks were compared pairwise using Bonferroni-Holmes-corrected Wilcoxon signed-rank tests. This analysis was performed to evaluate how the amount of feedback contributed to the control performance. Similarly, for the *Prosthesis tasks* the quality of force control (average MAE across levels 3 to 5) with and without explicit feedback was statistically compared using a Wilcoxon signed-rank test. To assess the force-control impact on individual levels, the same analysis was additionally performed separately for target levels 3 to 5.

## Results

### Scaling across target levels

Figure 3 depicts the distribution of the per-subject medians of the generated muscle activations and grasping forces across target levels in each task. For level 2 in the *Prosthesis task*, the subjects generated forces close to the target level while undershooting substantially with the myoelectric commands (compare Fig. 3a2, a3, b2 and b3), exploiting the coupling mechanism described in the section Experimental protocol. For level 6, the subjects often saturated the commands as well as the forces, as discussed above, resulting in (artificially) dense distributions and medians close to 100%. Generally, the results demonstrated that the subjects could successfully scale their generated muscle activations and/or forces according to the indicated target levels, even without any direct feedback on the controlled variable. In each task consistently (*p* < 0.05 in each Friedman test), the overall medians of the generated muscle activations and grasping forces significantly increased (*p* < 0.05 after Bonferroni-Holmes correction) from one target level to the next, for all the neighboring levels, clearly showing the subjects’ scaling ability. The range between the overall medians in the extreme levels was 66% for the *EMG task*, 88% in the EMG envelope and 74% in force for the *Prosthesis task without feedback,* and 76% in the EMG envelope and 71% in force for the *Prosthesis task with feedback*. The addition of the feedback sources seemed to have a beneficial effect on the scaling, as the dispersion of the subject-specific medians became lower, observable by comparing the interquartile ranges (the length of the boxplots), from Fig. 3a1 over a2 to a3 and from b2 to b3.

### Performance between tasks

**Fig. 4:**
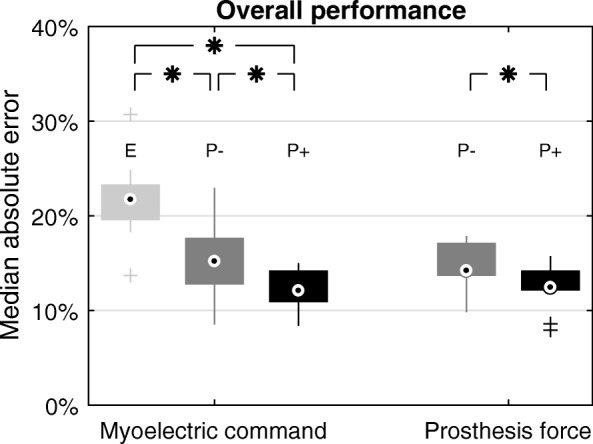
Overall performance in generating myoelectric commands and prosthesis forces for each task. For the *EMG task* (E, light grey), and the *Prosthesis task without* (P-, dark grey) and *with *(*p*+, black) *feedback*, the (per-subject) median absolute errors (MAEs), averaged across target levels 3 to 5, of the muscle activations and prosthesis forces are visualized using boxplots, depicting the median (circle), interquartile range (box), maximal/minimal values (lines) and outliers (pluses). Significant differences in MAE between the tasks are marked with an asterisk (*p* < 0.05, Bonferroni-Holmes-corrected in case of the myoelectric commands)

**Fig. 5:**
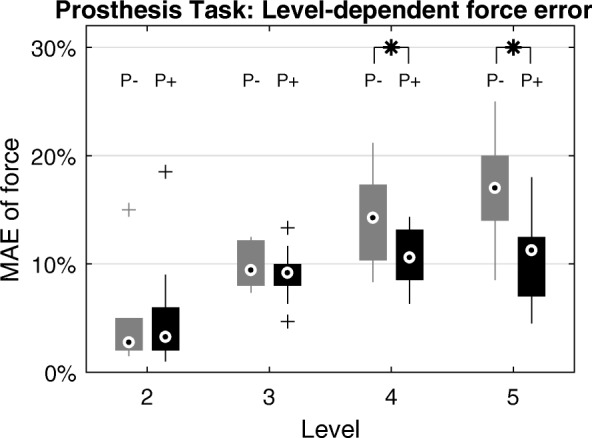
Performance in generating prosthesis forces for each analyzed target level. For the *Prosthesis task without* (P-, dark grey) and *with *(P+, black) *feedback*, the (per-subject) median absolute errors (MAEs) are visualized using boxplots, depicting the median (circle), interquartile range (box), maximal/minimal values (lines) and outliers (pluses). Significant differences in MAE between the tasks are marked with an asterisk (*p* < 0.05)

Figure 4 shows the overall MAE (across levels 3 to 5, as described in the Methods) for the generated muscle activations and grasping forces for each task, assessing the quality of myoelectric-command generation and prosthesis-force control between the tasks. The overall median MAE of the generated muscle activations was largest for the *EMG task* (22%), lower in the *Prosthesis task without feedback* (15%) and lowest in the *Prosthesis task with feedback* (12%). The pairwise differences were all statistically significant, as revealed by a Friedman test (*p* < 0.001) and significant post-hoc comparisons. Adding prosthesis motion and sound to proprioception significantly improved the command generation performance (*EMG task* vs. *Prosthesis task without feedback*), and providing explicit visual feedback improved the myoelectric control even further (*Prosthesis task without feedback* vs. *with feedback*). In the *Prosthesis task*, the improved myoelectric control also resulted in more accurate force generation, as the MAE of the generated forces decreased slightly but significantly when the explicit force feedback was introduced (Fig. 4, forces, 13% vs. 14% in the *Prosthesis task* with vs. without feedback). The quality of force control for each individual target level is presented in Fig. 5. The explicit feedback was not beneficial for level 3 but significantly improved force control at the higher levels (4 and 5, median improvement of 4% and 6%, respectively).

## Discussion

There have been no systematic studies so far assessing the baseline ability of human subjects to generate myoelectric commands and control a prosthesis force at a range of levels in “open-loop”, that is, with no explicit feedback on the grasp outcome. We have investigated incidental and explicit feedback in the context of prosthesis grasping. However, it is likely that the insights could be generalized to controlling other prosthesis functions. For example, the subjects might be able to control the wrist rotation speed (e.g., rotate slowly or quickly) using muscle proprioception and motor sound, without even looking at the hand. Finally, it should be emphasized that implicit and explicit feedback are components of the larger human motor control scheme that needs to be considered in its totality in order to properly model and understand closed-loop prosthesis control. This includes, among others, the ability of the subject to learn, predict, and adapt [19, 28–30], which is likely to have a substantial effect on the use and relevance of the feedback in general (either explicit or implicit).

Our results demonstrated that even in naïve users, the control without explicit feedback is not completely blind (open loop) but, on the contrary, surprisingly good. This could explain the unexpected outcome in some studies [13, 14, 25], where artificial feedback failed to provide any benefit. In other studies, the task was to produce few force levels (e.g., low, medium and high) [9, 12, 31] and the present experiment demonstrated that the subjects can already do more than that even with only minimal implicit feedback (muscle proprioception). Nevertheless, we argue that this type of feedback is useful for gross control tasks (force control during routine grasping); more delicate tasks, such as individual finger control in a dexterous prosthesis [32] or the use of prosthesis for haptic exploration [33], would still require artificial tactile feedback.

Looking at the *EMG task* only, this study shows that the feedback obtained through muscle proprioception is an important feature inherently available in myocontrol, essentially making myocontrol closed-loop control. This feedback source would not be present with other types of interfacing, such as nerve interfacing for motor commands without a target muscle. Using muscles for control is therefore very beneficial not just because they provide good control signals, but also because they provide very intuitive feedback.

The subjects could indeed generate five distinct levels, but they would not necessarily match the actual target levels, as the mean absolute error was between 10 and 20%. The quality of myoelectric control consistently improved with the addition of more feedback sources. Increasing the amount of implicit information, from proprioception only to proprioception and prosthesis motion and sound, and finally providing explicit feedback, steadily decreased the error in generating myoelectric commands. Interestingly, the improvement was more substantial when introducing the prosthesis (*EMG task* to *Prosthesis task without feedback*) compared to when adding explicit feedback on top of prosthesis motion and sound (*Prosthesis task without feedback* to *Prosthesis task with feedback*). The improvement in myoelectric control due to explicit feedback translated into better force control, but this benefit was modest overall.

The explicit force feedback significantly improved the performance when the uncertainty of myocontrol was high, since the EMG signals become more variable as the contraction level increases [34]. Overall, these results point to the importance of considering the implicit feedback when designing a closed-loop prosthetic system. In the present study, for example, the impact of increasing the implicit feedback on performance was more substantial than that of introducing the explicit information. It should also be considered that the provided explicit sensory feedback was ideal, being provided as continuous visual feedback. In reality, no feedback modality could provide such an accurate feedback on EMG or force in a real-life application. Therefore, the improvement observed here should be the very best that feedback can provide; yet, its benefit was rather small.

However, it should be noted that the controllability in the *Prosthesis task* was somewhat limited due to the inherent characteristics of a myoelectric prosthesis. The non-backdrivability, high impedance, and non-linear effects due to friction, especially while the prosthesis is closed around the object, might be a reason why the improvement in performance with the explicit feedback was limited. Effectively, this has prevented the subjects from exploiting the force feedback in real-time (i.e., within a single trial). Instead, the explicit feedback was used intermediately, on a trial-to-trial basis. The subjects used the feedback to assess the task outcome, and based on the discrepancy between the intended and generated force level, they adjusted their motor command in the next trial. Finally, although the two tasks in the present study have been performed using a Michelangelo hand prosthesis, the general mechanisms of prosthesis operation (proportionality, non-backdrivability) are common to most commercial systems and, therefore, allow us to interpret the study conclusions in a more general and broader relevance. Likewise, the first condition (*EMG task*), demonstrating the vital role of muscle proprioception during myocontrol, is fully device independent.

Generally, the good baseline performance (*Prosthesis task without feedback*) suggests that force-feedback interfaces require a reasonably good resolution (< 15%) in order to have a chance to provide a benefit (increased performance with respect to the baseline), especially at the higher force levels. This is valuable information and should be considered in the future design of feedback interfaces.

### Study limitations

In the present study, the forearm muscles have been used, since they are relevant for controlling hand prostheses. However, we believe that the subjects would be able to exploit the incidental feedback in a similar manner when using other muscles, e.g., upper arm (transhumeral prosthesis). Nevertheless, this needs to be confirmed experimentally, as the controllability (fine vs. gross movements) and proprioception might be somewhat different.

We only tested a homogenous group of naïve able-bodied subjects. Proprioceptive feedback from the muscles is also available to people with amputation, albeit somewhat different depending on the extent of amputation, shortening of muscles, and changes of attachment points. General skills as well as the interpretation and exploitation of the visual and auditory cues should be similar, and in addition, a person with amputation could exploit the incidental cues available through the prosthesis socket. Therefore, we expect that the results would not be significantly different in naïve persons with amputation, and even more so, the general conclusions regarding the importance of incidental feedback in prosthesis control. In experienced people with amputation, the baseline performance is likely to be even better than suggested here, due to extensive prosthesis use [35].

It would certainly be interesting to further compare performance with prosthesis users, considering also their age and level of physical activity, as these factors might influence muscle proprioception [36, 37] but also vision and audition, which deteriorate with age.

Next, the present study has been purposefully conducted in well controlled laboratory conditions to obtain isolated insights into different feedback components, as explained in *Introduction*. Therefore, there was no background noise that could mask the prosthesis sounds, the hand was always clearly visible, and the task consisted of repeatable grasping of the same object. These conditions might facilitate the reliance on implicit feedback. In daily life, however, users will most likely encounter more challenging circumstances, which could make explicit feedback more important. For example, in our recent study, we have demonstrated that explicit feedback becomes relevant when the task complexity increases [38]. Hence, it is all the more important to validate our findings in amputees.

## Conclusion

This study provides a first systematic investigation of how implicit (proprioception, prosthesis sound, vision of the prosthesis) and explicit feedback (visual force information) interact to influence the performance of grasping force control. We showed that muscle proprioception alone already allows to scale the grasping forces. Adding the incidental feedback from the prosthesis improved the accuracy of this scaling by decreasing the deviations from the target levels. Importantly, the improvement was more substantial when increasing the amount of incidental feedback than when the explicit force feedback was introduced. These insights emphasize that a myoelectric interface already provides closed-loop control with good performance. Therefore, artificial feedback needs to be carefully designed to outperform this baseline and become truly effective.
